# Effects of Selection to Diflubenzuron and *Bacillus thuringiensis* Var. *Israelensis* on the Overwintering Successes of *Aedes albopictus* (Diptera: Culicidae)

**DOI:** 10.3390/insects12090822

**Published:** 2021-09-13

**Authors:** Charalampos S. Ioannou, Christos Hadjichristodoulou, Varvara A. Mouchtouri, Nikos T. Papadopoulos

**Affiliations:** 1 Laboratory of Hygiene & Epidemiology, Faculty of Medicine, School of Health Science, University of Thessaly, 41222 Larissa, Greece; ioannoubabis@yahoo.com (C.S.I.); xhatzi@med.uth.gr (C.H.); mouchtourib@uth.gr (V.A.M.); 2 Laboratory of Entomology & Agricultural Zoology, Department of Agriculture Crop. Production and Rural Environment, School of Agricultural Sciences, University of Thessaly, 38446 Volos, Greece

**Keywords:** Asian tiger mosquito, eggs diapause, insecticides efficacy, mosquito control

## Abstract

**Simple Summary:**

*Aedes albopictus* is an invasive mosquito species that is well established in many parts of Europe and poses high risks of autochthonous transmission of chikungunya and dengue viruses. The high dependency on commonly used larvicides such as Diflubenzuron (DFB) and *Bacillus thuringiensis* var. *israelensis* (*Bti*) to control its populations raise concerns for resistance development. Although studies addressing the potential for development of resistance in *Ae*. *albopictus* against these two important larvicides are essential for planning control programmes, no such data are available. Here, by imposing an increasing selective pressure to DFB and *Bti* over nine successive generations on a recently laboratory established *Ae*. *albopictus* population we determined the subsequent resistance levels and corresponding overwintering success of the selected populations relative to control (colonies that received no selection). Our findings revealed a moderate and a minor increase on the resistance levels following selection with DFB and *Bti,* respectively. No significant differences were observed regarding the overwintering successes between the two selected populations and the control, which suggests that the selected individuals retain an equal ability to persist in the wild on an annual base.

**Abstract:**

*Aedes albopictus* is an invasive mosquito species responsible for local transmission of chikungunya and dengue viruses in Europe. In the absence of available treatments, insecticides-based control remains one of the most important viable strategies to prevent emerging problems. Diflubenzuron (DFB) and *Bacillus thuringiensis* var. *israelensis* (*Bti*) are among the most commonly used larvicides for *Ae*. *albopictus* control with consequent concerns for the potential development of resistance. Studies on the resistance emergence in *Ae*. *albopictus* and its persistence in the wild to both DFB and *Bti* are essential for the efficient and sustainable planning of the control programmes. In this context, larvae from a recently laboratory established population were subjected to increasing selective pressure for nine successive generations using both DFB and *Bti*. The resistance levels and the overwintering success of the selected populations relative to control (colonies that received no selection) were determined. Results revealed an 8.5- and 1.6-fold increase on the resistance levels following selection with DFB and *Bti,* respectively. The selection process to both larvicides had no apparent impacts on the overwintering capability relative to control, suggesting the successful persistence of the selected individuals in the wild on an annual base.

## 1. Introduction

*Aedes albopictus* Skuse (Diptera: Culicidae) also known as the Asian tiger mosquito is classified as one of the 100 most invasive species in the world [1]. During the last 50–60 years, largely facilitated by human activities and climate change, it has extended its geographic distribution from its native range in tropical and subtropical Southeast Asia to all over the world [2]. Successful invasion and colonization of the northern temperate areas have been attributed to the remarkable ecological and physiological plasticity of this species [3]. In temperate regions, *Ae. albopictus* overwinters at the egg stage containing pharate first instar larvae in facultative diapause that occurs when maternal females experience short day photoperiod as pupae and adults [4]. In Europe, *Ae. albopictus* was recorded for the first time in Albania in 1979 [5], however awareness only spread in the early 1990s after the discovery of established populations in Italy [6]. Recently, it has displayed a wide distribution in both the Mediterranean basin and central Europe having established itself in at least 19 countries [7]. Due to its aggressive biting behavior, *Ae. albopictus* causes important nuisance problems that negatively affect the welfare levels while they may also act as an important discouraging factor for the tourism industry [8]. Moreover, as a competent vector of more than 32 arboviruses, including chikungunya and dengue, *Ae. albopictus* poses a major threat for public health [9]. In Europe, its presence has been associated with autochthonous transmission of dengue in Croatia (2010), France (2010, 2013–2015), and Spain (2014–2016) and of chikungunya in Italy (2007 and 2017) and southeastern France (2010 and 2014) [10]. Furthermore, as a consequence of its highly opportunistic feeding behavior on different animal species, *Ae. albopictus* has the potential to act as a bridge-vector of zoonotic pathogens from animal-to-animal and from animal-to-human [11].

As there no available drug treatments, *Ae. albopictus*-borne diseases such as dengue and chikungunya are managed mainly through vector control [12]. In recent years, new control approaches have been proposed including the Sterile Insect Technique (SIT), the Release of Insects carrying a Dominant Lethal (RIDL), the release of *Wolbachia*-infected mosquitoes and pyriproxyfen autodissemination, i.e., a self-delivery technique that manipulates mosquitoes’ behavior to carry and disseminate the killing agent (the IGR insecticide pyriproxyfen) to potential breeding sites [13]. Despite the encouraging results, certain limitations prevent the wide application of these approaches in the field. Among others, the main limitations in the area-wide application of SIT are the high production and transportation costs of sterilized males required to achieve successful suppression of the wild mosquito populations [14]. On the other hand, the implementation of both RIDL and *Wolbachia*-based strategies are under strict prohibition in many countries including the European Union due to environmental/ecological concerns, whereas the efficacy of pyriproxyfen autodissemination highly depends on the availability of breeding sites in a given area [13]. Therefore, conventional spray applications of insecticides still remain the principal mean for dealing with *Ae. albopictus* related problems.

In general, insecticide interventions prioritize larval over adult control, due to the higher anticipated efficacy and the lower human health and environmental risks. Currently in Europe, larval control relies almost exclusively on the Insect Growth Regulator (IGR) Diflubenzuron (DFB) and the microbial control agent *Bacillus thuringiensis* var. *israelensis* (*Bti*) [15]. DFB is a chitin biosynthesis inhibitor that interrupts insects’ normal development during the immature stages [16]. During sporulation *Bti* produces a mixture of Cry (Cry11Aa, Cry4Ba, Cry 4Aa and Cry10Aa) and Cyt (Cyt1Aa and Cyt2Ba) toxins [17]. The Cry toxins act synergistically with the Cyt by disrupting the integrity of the larvae midgut membrane [18,19]. The synergistic interactions between the Cyt and the Cry toxins are considered the key factor for the drastic enhancement of the larvicidal activity of the later (Cry toxins) [20] as well as for the delay and the low risk of resistance selection to *Bti* in mosquitoes [17,21].

The intensive use of both DFB and *Bti* for mosquitoes’ control pose potential risks for the development of resistance. While a recent study suggests their suitability for the effectiveness control of *Ae. albopictus* [22], findings regarding other species do not fully confirm this point of view. In particular, high levels (up to 128-fold) of DFB resistance associated with specific mutations in the Chitin synthase gene have been identified in wild *Cx. pipiens* populations from different countries with a high focal distribution in areas characterized by intensive applications of this product for both mosquitoes and agricultural pests control [23,24,25]. On the other hand, there is only a single record to support for substantial (33-fold) *Bti* resistance development in *Cx. pipiens* mosquito natural populations [26]. Despite these findings, no efforts have been made so far to explore the potential resistance development in *Ae. albopictus* to DFB and *Bti*.

Insecticide resistance development is often accompanied with significant fitness costs as a result of trade-offs associated with the function of the resistance mechanisms [27]. Moreover, adverse abiotic conditions may largely affect the expression of these costs in mosquitoes [28]. In this regard, winter in temperate regions represents a very challenging season for the survival of mosquitoes and therefore potential insecticide resistance associated fitness costs may reduce their overwintering capacity. The ability of the insecticide tolerant/resistant individuals to survive during the cold period is crucial for both the stability and evolution of acquired resistance in the wild from year to year. While the effects of DFB and *Bti* selection on the overwintering success of the medically important mosquito species *Cx. pipiens* have recently been elucidated [28], no such information is available for *Ae. albopictus* given its different winter biology. Considering the above mentioned knowledge gaps regarding the potential performance of *Ae. albopictus* to DFB and *Bti* extensive exposure, the aim of the present study was to explore how selection of both larvicides affects the resistance development as well as the overwintering success of this species.

## 2. Materials and Methods

### 2.1. Mosquitoes Used and Rearing Methods

An *Ae. albopictus* population was established during early September to mid-October of 2017 from approximately 2000 eggs that were collected using a network of 20 ovitraps set up at the vicinity of Volos (39°21′39.71″ N, 22°56′32.93″ E) and Larisa (39°38′12.80″ N, 22°25′3.40″ E) city, Thessaly county, Greece. Over the past 10 years, regional mosquito control interventions, including the collection sites, are mainly based on the routine use of DFB and *Bti* [28]. The ovitraps consisted of glossy black, cylindrical (15 cm high and 11 cm diameter) plastic pots filled with 800 mL tap water up to an overflow hole. Ten wooden strips (tongue depressors of 15 × 1.8 cm^2^) with one side properly scratched by a serrated knife, were attached with paper clips vertically in the inner of each pot serving as oviposition substrates. Strips from each ovitap were collected every 8–10 days, placed in plastic sealing bags and transferred to the laboratory. Mosquitoes’ colonization took place under standard conditions (25 ± 1 °C, 65 ± 5% relative humidity and a photoperiod of 14L:10D with a simulated dusk and dawn for 45 min). Upon adult emergence, specimens’ identification was performed using a dichotomous key [29] to avoid possible colony contamination by the relative *Ae. cretinus* species, which is also present in some regions of Greece but at extremely low densities [22]. Larvae and adult rearing procedures of the established population were identical of that described for *Cx. pipiens* by Ioannou et al. [28]. In brief, groups of approximately 1000 larvae were reared inside plastic containers provided with 3 L of water and 2 g of dry cat food while adults were maintained in 32 × 32 × 32 cm^3^ screened cages at a density of 600–700 individuals having constant access to 10% sugar solution. Mosquitoes were offered an arm feeding by a volunteer (C. Ioannou) until all responding females (usually 80–90% of total) in each cage completed a full blood meal. Females had access to oviposit on moist filter papers submerged in 500 mL of tap water inside black, plastic cylindrical containers. Before the initiation of any experimental procedure, the mosquito population was reared for three generations to establish a uniform genetic background.

### 2.2. Larval Bioassays

The susceptibility of the established *Ae. albopictus* population was evaluated against technical grade DFB (Purity ≥ 99.8%, Pestanal^®^, Sigma-Aldrich, Taufkirchen, Germany) and formulated *Bti* (Vectobac^®^ 12AS, 11.61% *w*/*w Bti* serotype H-14, strain AM65-52, 1200 ITU/mg, Valent BioSciences Corporation, Libertyville, IL, USA) following the standard WHO guidelines [30]. Stock solutions were prepared in 99.5% acetone for DFB and distilled water for *Bti* and stored at –22 °C until use for a maximum of two weeks before being replaced by new ones. Bioassays for DFB and *Bti* were performed using late third and early fourth instar larvae, respectively. Each time, 25 larvae were transferred into plastic cups provided with 99 mL of distilled water and 1 mL of larvicide solution at the desired concentration. Control cups received 1 mL of acetone and distilled water in the case of DFB and *Bti,* respectively. Due to the slow mode of DFB action, in this case larvae were provided with cat food powder at a concentration of 100 mg/L to allow development. Six and five concentrations at the mortality range 10–95% were used for DFB and *Bti* bioassays. For each concentration and the respective controls six replicates (cups with 25 larvae) were performed. Bioassays were repeated three times on different days using new batches of larvae and larvicide solutions. Larval mortality for *Bti* was recorded after 24 h while determination of adult emergence inhibition for DFB was completed when all larvae in the control cups emerged as adults or died. EI_50_, EI_80,_ and EI_90_ values (EI: Adult emergence inhibition) for DFB and LC_50_, LC_80,_ and LC_90_ values (LC: Lethal concentration) for *Bti* were estimated using log-probit analysis [31].

### 2.3. Larval Selection

Larvae were selected for six successive generations to fixed IE_80_ and LC_80_ concentrations of DFB and *Bti,* respectively. At this point, the induced susceptibility levels were recalculated and the selection process was continued for three additional generations by applying the new IE_80_ and LC_80_ fixed doses. The selection process was identical of that described for *Cx. pipiens* by Ioannou et al. [28]. In brief, groups of approximately 1000 larvae, of appropriate developmental stage, were placed into rearing containers provided with 3 L of water and the fixed doses, while the resulting adults were reared following standard procedures. During the selection process, two additional larvae groups (≈1000 individuals each) were manipulated in exactly the same way but in the absence of DFB and *Bti* exposure serving as the control population. At the end of the selection process (F9 generation), new bioassays were performed to establish the induced resistance levels against both DFB and *Bti*. Moreover, the winter survival of diapausing eggs originating from each selected population and the control was determined.

### 2.4. Winter Survival

To induce *Ae. albopictus* females from each experimental population to lay diapausing eggs, both immature and adult stages were reared in an environmental chamber set at 20 °C, 8L:16D, and 70% RH [32]. Seven to 10 days after adult emergence, females were offered a blood meal (arm feeding). Soon after, 200 blood fed females from each population were allocated in 2 rearing cages (100/cage) and allowed to lay their eggs for 15 days in 2 ovitraps (one/cage), each containing 15 wooden strips. Ovitraps were then removed from the cages and held for 7 days inside the environmental chamber to permit embryonation. Then, eggs were cold acclimated for a week inside a dark incubator set at 10 ± 1 °C and RH 75 ± 5% to enhance their cold hardiness [4]. Before being exposed outdoors, each wooden strip was pictured under a binocular stereoscope (ZEISS, SteREO, Discovery.V12, Oberkochen, Germany) equipped with a digital camera (ZEISS, AxioCam, ERc 5s, Göttingen, Germany) to facilitate eggs counting. Then, strips were clipped to the inner edge of ovitrap pots with a single drainage hole placed 4.5 cm from the bottom. This configuration allowed ambient rain water to remain and moisten the wooden strips inside the pots but not flood the eggs. Two such pots were considered for each experimental population hosting a total of 30 wooden strips (15 each). Pots were covered, including the drainage hole, with a mesh screen (mesh size 1.0 mm) and fitted randomly side by side into a black, plastic, mesh box (mesh size 1 cm). To prevent access of macroscopic organisms, a second box was fastened on the top serving as a cover. Pots were positioned in the corner of a concrete wall at the University facilities, well protected from both direct sun light and strong winds. Eggs exposure took place on 12 November 2019. An outdoor data logger (HOBO Pro v2, Onset Computer Corporation, Bourne, MA, USA) set to receive 4 recordings of both temperature and RH per 24 h was attached at the bottom plastic box that hosted the pots. Based on empirical evidences suggesting the first appearance of *Ae. albopictus* adults in the area at the beginning of May overwintered egg strips were collected on 10 April 2020 and transferred to the laboratory. Strips were again pictured under the stereoscope and intact eggs were counted. To stimulate larval hatching, each egg strip was submerged for a week in a white, plastic container with 300 mL stale tap water provided with 0.05 g of cat food. After that, egg strips were removed from the water and left to dry for 72 h before a subsequent flooding was performed for another week. This procedure was repeated four times, until no more hatching events were observed, while the remaining unhatched eggs were considered dead. For each experimental population, survival of overwintered eggs under the ambient natural conditions was determined.

### 2.5. Statistical Analysis

Dose-response larval bioassays were subjected to Probit Analysis by transforming dose to log after corrections for control mortality according to Abbott’s formula [33]. Emergence inhibition (EI) and lethal concentrations (LC) values for DFB and *Bti,* respectively, where considered significantly different when 95% of confidence limits (CL) failed to overlap (*p* < 0.01). For the two selected populations and the control, the numbers of intact eggs in wooden strips before and after outdoor exposure and the respective proportions of hatched larvae were determined. Female fecundity and overwintered eggs hatchability data were analyzed using one-way analysis of variance after appropriate transformations for normality and homoscedasticity when necessary followed by Tukey’s HSD post hoc to separate means (*p* < 0.05). Data analysis was performed using IBM SPSS 25 (IBM Corp., Armonk, NY, USA).

## 3. Results

**Figure 1:**
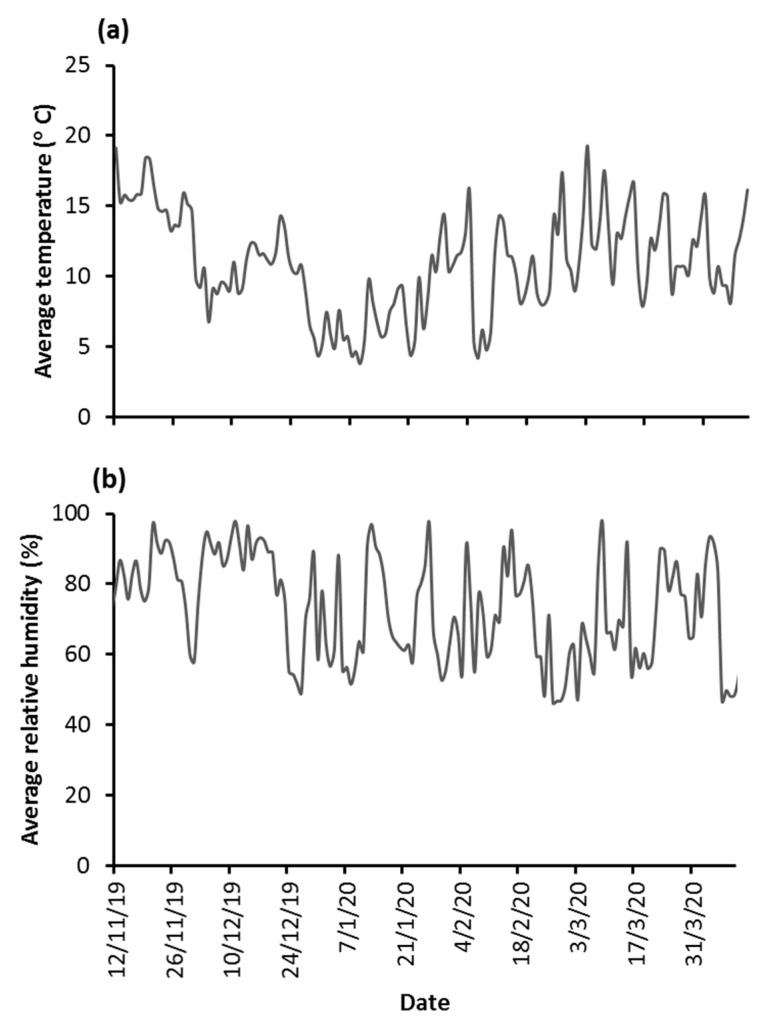
Temperature (**a**) and humidity (**b**) profiles at the study site during winter 2019/2020.

Larval bioassay results are given in Table 1 and Table 2 for DFB and *Bti,* respectively. The selection process with DFB for six successive generations by applying the EI_80_ corresponding dose, as determined for the established population, resulted in 3.6 and 4.4 Resistance Ratio (RR) values relative to the control as far as EI_50_ and EI_90_ are concerned. Selection for three additional generations with the new established EI_80_ corresponding dose, as determined at the end of the previous selection process (F6 generation), nearly doubled these values (Table 1). In contrast, selection to *Bti* had a little impact on the observed susceptibility levels (Table 2). Selection against both DFD and *Bti* reduced female fecundity relative to the control, however no significant differences were observed (Table 3). Recovery rates of diapaused eggs after outdoor exposure reached more than 95% for all tested populations (Table 3). Ambient temperature and relative humidity data during eggs’ overwintering are depicted in Figure 1. Temperatures ranged between 3.8 and 19.3 °C with a mean value of 10.9 °C indicating a rather mild cold period. Selection to *Bti* significantly increased the survival rates of overwintered eggs relative to control and the DFB selected population (Table 3). Moreover, the hatching rates of viable eggs (i.e., those that yielded larvae) following the first immersion in the water were significantly higher in the two selected populations relative to control.

## 4. Discussion

Despite the recent advances in new management approaches, the control of *Ae. albopictus* still relies to a great extent on the utilization of insecticides, raising concerns over resistance development. Understanding both the establishment and evolution of acquired resistance in the wild is crucial for the sustainability of management programmes. In this context, the current study represents a detailed investigation on the resistance development and establishment against two widely used larvicides. By applying an increasing selective pressure with DFB and *Bti* to *Ae. albopictus*, over a relative small number of successive generations, we found a moderate and a minor decrease on the susceptibility levels, respectively. Interestingly, selection to both larvicides had no apparent fitness costs in terms of female fecundity and the winter survival of diapausing eggs relative to control (non-selected population), suggesting an equal ability of the selected individuals to persist in the wild. These findings provide important insights on the potential development of resistance in *Ae. albopictus* against these commonly used insecticides, as well as on its prevalence in the wild from year to year.

The EI_50_ value determined for the established *Ae. albopictus* population (0.0017 mg/L) in the current study is remarkably lower than the WHO recommended DFB concentration (0.25 mg/L) for potable water [34] as well as the respective EI_50_ value (0.376 mg/L) reported for field collected *Ae. albopictus* populations from southern Switzerland [12]. In support of our findings, a recent survey revealed 100% mortality of *Ae. albopictus* populations collected from different regions of Greece at DFB doses below 0.02 mg/L, suggesting also high susceptibility [22]. On the other hand, the estimated LC_50_ value for *Bti* was 4.8 to 7.5-fold lower compared to the respective values recorded for other Greek populations of this species according to the previously mentioned study. However, such variations are not uncommon as an earlier survey in the island of Cyprus revealed an over 10-fold range in *Bti* LC_50_ values for wild collected mosquito populations of *Cx. pipiens* [35]. Interestingly, the LC_50_ value determined in our study is less than reported for laboratory strains of *Ae. albopictus* [36,37] suggesting high susceptibility to *Bti*.

Despite the high DFB susceptibility of the established population, the selection process resulted in the development of moderate resistance levels. During the first six successive generations of selection to the fixed dose corresponding to EI_80_, only a slight increment on the RR_50 and 90_ values was observed. This is in line with the results obtained by Belinato and Valle [38] who applied exactly the same selection protocol on *Aedes aegypti*. However, selection for three additional generations with the new established EI_80_ corresponding dose nearly doubled these values, indicating that the resistance development and evolution to DFB is highly facilitated by the imposition of increasing selective pressure. Thus, it appears that under certain conditions, even highly susceptible populations of *Ae. albopictus* as in our case, are capable of developing appreciable resistance levels. Recent evidences suggest the high suitability of DFB for the Asian tiger mosquito control [22], and this is partially true for other medically important mosquito species. In particular, very high DFB resistance levels have already been detected in wild populations of *Cx. pipiens* [23,39]. Moreover, subsequent surveys revealed a high focal distribution of the resistant individuals in areas with a strong background of intensive DFB use [24]. Therefore, in the light of our findings, the possibility of similar DFB resistance patterns cannot be excluded in the case of *Ae. albopictus*, highlighting the need for systematic resistance surveillance, especially in high risk areas.

On the other hand, selection to *Bti* had a negligible impact on the resistance levels of the *Ae. Albopictus*-established population. With the exception of a single record in *Cx. pipiens* [26], there are no consistent data to support for substantial *Bti* resistance development in mosquitoes [40]. As previously mentioned, the synergistic interactions between the Cyt and the Cry toxins are considered the key element for both the delay and the low risk of resistance selection to *Bti*. Conversely, extensive exposure to single Cry toxins can result in substantial resistance [41,42,43]. Another interesting aspect that appears to facilitate the low risk of *Bti* resistance development following extensive selection, is the fact that in the absence of selective pressure, any acquired resistance declines rapidly within a few generations [44,45]. While the reasons underlying this phenomenon remain largely unknown, the related high fitness costs have been proposed as a possible explanation [45].

Although, selection against both DFD and *Bti* reduced female fecundity compared to control, no significant differences were observed, suggesting a moderate fitness cost. In contrast to our findings, selection of *Cx. pipiens* and *Ae. aegypti* for 20 and 6 successive generations to *Bti* and DFB, respectively, resulted in a significant decrease of female fecundity [38,44]. However, it should be stressed that in these studies, tested females were previously exposed during the larval stage to the respective insecticides. Therefore, these results indicate primarily delayed effects following larvae exposure to sub-lethal doses of *Bti* and DFB rather than actual fitness costs. On the other hand, the females in our study that laid the diapausing eggs had no prior contact as immatures to both larvicides and thus we are confident that their oviposition output reflects the net costs induced as a result of the imposed selective pressure.

Survival of overwintering *Ae. albopictus* eggs under natural conditions is highly affected by the exposure methods. In this regard, our exposure configuration resulted in a 60% average hatching rate of the control population eggs, which is almost identical with the highest rate determined in a recent study that assessed different overwintering exposure protocols [46]. Selection to DFB had no apparent effect on the hatching rates of the overwintered eggs relative to control, suggesting no differences in their winter physiology. However, most importantly, it means that the progenies of the DFB selected individuals have the same survival prospects as the controls, providing the background for further resistance development in the coming year. In this context, the appearance of resistance without evidence of a trade-off in overwintering survival may pose a major challenge to future management of *Ae. albopictus* with DFB. Interestingly, while selection against *Bti* had a minor impact on the susceptibility levels, it conferred a moderate, though significant advantage regarding the overwintering success of the tested population relative to control. To the best of our knowledge this is the first report of a potential advantage associated with *Bti* selection in mosquitoes. Similarly to our findings, a positive association between selection to *Bacillus thuringiensis* subsp. *kurstaki* and the overwintering survival of the agricultural pest *Trichoplusia ni* (Lepidoptera: Noctuidae) has been recorded [47]. Authors attributed this phenomenon to possible pleiotropic effects according to which an allele responsible for resistance is positively influencing one of the components related with increased winter survival. However, this justification is not valid in our case as there are no consistent recordings of mosquito resistance to *Bti,* as mentioned above, and therefore no such alleles have been described for any mosquito species so far. As has emerged from the current study, selection to *Bti* resulted in a lower fecundity output relative to the other two tested populations (control and DFB selected). Therefore, it is possible that a greater maternal investment in terms of eggs nutrition occurred in this case, providing the background for a better overwintering capacity.

In the present study, the vast majority of eggs from all tested populations hatched after the first inundation followed by subsequent minimal hatchings. This is in line with an earlier study where overwintered eggs also went into a first mass hatching followed by other minor ones [46]. *Ae. albopictus* as well as other species of the genus *Aedes*, has a distinctive hatching pattern known as installment hatching. According to this, some eggs may hatch readily following inundation, while others may remain dormant for varying periods despite being underwater [48]. Selection to both DFB and *Bti* resulted in a higher synchronization of larvae hatching during the first inundation relative to control. This is most likely because the selection process in both cases enabled the prevalence of individuals with this attribute. While the biological significance of this finding remains largely unknown, at least theoretically it could promote the co-occurrence of the selected individuals during spring, facilitating their interbreeding and therefore the establishment of resistant/tolerant populations in the wild.

## 5. Conclusions

By using an *Ae. albopictus* population with high, inherent susceptibility to both DFB and *Bti* we showed that exposure to progressively increasing selective pressure resulted in a moderate and a minor increase of the resistance levels, respectively. Moreover, selection of both larvicides revealed no significant trade-offs on females’ fecundity as well as on the overwintering success of their descendants. These findings provide important insights regarding the potential risks of resistance development in *Ae. albopictus* against these two widely used larvicides as well as on its persistence in the wild on an annual base. Based on this, systematic monitoring for potential resistance development is imperative in order to maintain the current effectiveness of these two larvicides against *Ae. albopictus* populations and especially of DFB.

## Figures and Tables

**Table 1:** Emergence inhibition (EI) values of diflubenzuron (DFB) against *Ae. albopictus* before and after the selection process for six and nine successive generations, respectively.

Population	N *	EI_50_ (95% CL) ^a^	RR_50_	EI_90_ (95% CL) ^a^	RR_90_	Slope	*X*^2^ (df)
Pre-selection							
Colony	3150	0.0017 (0.0014–0.0019)	–	0.0039 (0.0036–0.0041)	–	3.56	119.24 (105)
Post-selection							
Control	2700	0.0022 (0.0016–0.0026)	–	0.0041 (0.0037–0.0045)	–	4.68	156.25 ^b^ (87)
F6 DFB	2700	0.0080 (0.0070–0.0089)	3.6	0.0182 (0.0170–0.0196)	4.4	3.58	62.23 (87)
Control	2700	0.0022 (0.0019–0.0025)	–	0.0040 (0.0038–0.0043)	–	4.86	99.94 (87)
F9 DFB	2700	0.0188 (0.0174–0.0200)	8.5	0.0301 (0.0288–0.0319)	7.5	6.28	24.73 (87)

* Number of larvae tested. ^a^ EI values are expressed in milligrams per liter, and they are considered significantly different when 95% of confidence limits (CL) fail to overlap (*p* < 0.01). ^b^ Since goodness-of-fit test is significant (*p* < 0.05), a heterogeneity factor was used in the calculation of confidence limits (CL).

**Table 2:** Lethal concentration (LC) values of *Bti* against *Ae. albopictus* before and after the selection process for six and nine successive generations, respectively.

Population	N *	LC_50_ (95% CL) ^a^	RR_50_	LC_90_ (95% CL) ^a^	RR_90_	Slope	*X^2^* (df)
Pre-selection							
Colony	2700	0.027 (0.024–0.034)	–	0.051 (0.047–0.056)	–	4.59	94.44 (87)
Post-selection							
Control	2700	0.032 (0.030–0.034)	–	0.046 (0.044–0.050)	–	8.08	132.02 ^b^ (87)
F6 *Bti*	2700	0.041 (0.031–0.048)	1.3	0.067 (0.062–0.071)	1.5	6.04	59.86 (87)
Control	2700	0.030 (0.028–0.032)	–	0.045 (0.043–0.047)	–	7.70	58.39 (87)
F9 *Bti*	2700	0.047 (0.036–0.055)	1.6	0.083 (0.076–0.088)	1.8	5.20	80.62 (87)

* Number of larvae tested. ^a^ LC values are expressed in milligrams per liter, and they are considered significantly different when 95% of confidence limits (CL) fail to overlap (*p* < 0.01). ^b^ Since goodness-of-fit test is significant (*p* < 0.05), a heterogeneity factor was used in the calculation of confidence limits (CL).

**Table 3:** Female fecundity and winter larvae survival parameters of *Ae. albopictus* diapausing eggs originated from populations that either were selected against diflubenzuron (DFB) and *Bti* for nine successive generations or not (control). For each population, 200 blood fed females were allowed to lay their eggs in 30 wooden strips.

	Mean ± SE			
Population	Eggs/Wooden Strip Before Outdoor Exposure	Eggs/Wooden Strip after Outdoor Exposure	Hatching Rates of Recovered Eggs%	Hatching Rates of Viable Eggs% during the First Water Immersion
Control	304.47 ± 23.70 ^a^	294.10 ± 23.14 ^a^	59.98 ± 1.31 ^b^	85.50 ± 1.80 ^b^
F9 DFB	279.63 ± 17.36 ^a^	273.57 ± 17.20 ^a^	60.15 ± 1.35 ^b^	91.71 ± 0.87 ^a^
F9 *Bti*	266.47 ± 25.07 ^a^	257.60 ± 24.73 ^a^	66.17 ± 1.96 ^a^	93.35 ± 1.32 ^a^
*F*	0.75	0.73	5.05	8.96
df	2.87	2.87	2.87	2.87
*P*	0.476	0.485	0.008	<0.0001

Means in the same column followed by the same letter indicate no significant differences (Tukey’s HSD test, *p* > 0.05).

## Data Availability

The data presented in this study are available on request from the corresponding author.
